# Carbon black nanoparticles induce type II epithelial cells to release chemotaxins for alveolar macrophages

**DOI:** 10.1186/1743-8977-2-11

**Published:** 2005-12-06

**Authors:** Peter G Barlow, Anna Clouter-Baker, Ken Donaldson, Janis MacCallum, Vicki Stone

**Affiliations:** 1 M.R.C/University of Edinburgh Centre for Inflammation Research, Queen's Medical Research Institute, 47 Little France Crescent, Edinburgh EH16 4TJ, UK; 2 Biomedicine Research Group, Napier University, 10 Colinton Road, Edinburgh EH10 5DT, UK; 3 ELEGI/Colt Laboratories, M.R.C/University of Edinburgh Centre for Inflammation Research, Queen's Medical Research Institute, 47 Little France Crescent, Edinburgh EH16 4TJ, UK

## Abstract

**Background:**

Alveolar macrophages are a key cell in dealing with particles deposited in the lungs and in determining the subsequent response to that particle exposure. Nanoparticles are considered a potential threat to the lungs and the mechanism of pulmonary response to nanoparticles is currently under intense scrutiny. The type II alveolar epithelial cell has previously been shown to release chemoattractants which can recruit alveolar macrophages to sites of particle deposition. The aim of this study was to assess the responses of a type II epithelial cell line (L-2) to both fine and nanoparticle exposure in terms of secretion of chemotactic substances capable of inducing macrophage migration.

**Results:**

Exposure of type II cells to carbon black nanoparticles resulted in significant release of macrophage chemoattractant compared to the negative control and to other dusts tested (fine carbon black and TiO_2 _and nanoparticle TiO_2_) as measured by macrophage migration towards type II cell conditioned medium. SDS-PAGE analysis of the conditioned medium from particle treated type II cells revealed that a higher number of protein bands were present in the conditioned medium obtained from type II cells treated with nanoparticle carbon black compared to other dusts tested. Size-fractionation of the chemotaxin-rich supernatant determined that the chemoattractants released from the epithelial cells were between 5 and 30 kDa in size.

**Conclusion:**

The highly toxic nature and reactive surface chemistry of the carbon black nanoparticles has very likely induced the type II cell line to release pro-inflammatory mediators that can potentially induce migration of macrophages. This could aid in the rapid recruitment of inflammatory cells to sites of particle deposition and the subsequent removal of the particles by phagocytic cells such as macrophages and neutrophils. Future studies in this area could focus on the exact identity of the substance(s) released by the type II cells in response to particle exposure.

## Background

Exposure to the combustion-derived nanoparticulate component of particulate matter (PM) has been implicated in adverse effects in toxicology [1-3] and epidemiology [4-9] studies. Nanoparticles have at least one dimension less than 100 nm, similar to ultrafine particles which have a diameter of less than 100 nm. Nanoparticles have been reported to display increased toxicity compared to an equivalent mass of fine particles of the same material when inhaled into the rat lung [10]. This has been attributed to the oxidative stress emanating from the greater surface area of the nanoparticles [11-13] and their potential to interact with other components of PM such as transition metals [12].

Macrophages are the primary defence against inhaled particulates on the airspace surfaces. Following particle deposition, macrophages phagocytose particles and transport them out of the lung via the mucociliary escalator (MCE). It is hypothesised that the type II epithelial cells in the alveolar space play an important role in the modulation of inflammatory processes within the lung. Studies have shown that type II alveolar epithelial cells can release inflammatory cytokines such as rantes [14], monocyte chemoattractant protein-1 (MCP-1) [14], interleuklin-8 (IL-8) [16], tumour necrosis factor alpha (TNFα) [15] and the complement protein C5a [17] which can recruit leukocytes to sites of inflammation by acting as chemoattractants [16,17].

Driscoll *et al*., [18] demonstrated that α-quartz can induce an increase in MIP-2 mRNA in type II cells which could contribute to the accumulation of neutrophils seen in quartz-exposed lungs. Driscoll *et al*., [15] has also demonstrated that TNFα, produced by macrophages exposed to particles, can induce type II cells to release chemokines such as MIP-2. Jimenez *et al*., [19] showed the same effect in macrophages exposed to PM_10_. Barrett *et al*., [20] reported that the production of MIP-2 and MCP-1 by a murine type II cell line following silica exposure was mediated by TNFα which, together with silica exposure, could also induce ROS production by the type II cells. Becher *et al*., [21] also demonstrated that release of type II cell IL-6, TNFα and MIP-2 differed depending on the type of stone particle that the cells were exposed to.

O'Brien *et al*., [14] showed that macrophages migrated towards conditioned medium obtained from type II cells treated with IL-1α. Chemokines in the conditioned medium such as rantes, MCP-1 and Granulocyte Monocyte Colony Stimulating Factor (GM-CSF) were the agents responsible for the chemotaxis. Stimulation of type II cells with bradykinin has also been shown to induce the production of proteins that are chemotactic for neutrophils and monocytes [22]. Paine III *et al*., [23] observed increased MCP-1 production by type II cells stimulated with IL-1 and TNFα. We have previously shown that carbon black nanoparticles can activate serum to produce factors that induce macrophage migration [24].

This manuscript describes a study in which two cell lines are used as a model to examine the effects of fine and nanoparticle forms of both carbon black and titanium dioxide on potential of type II cells to generate substances that induce macrophage migration. We hypothesised that exposure of a type II cell line to fine and nanoparticles results in the release of chemotactic proteins from the type II cells. We further hypothesise that, in the mammalian lung, these proteins may play an important role in the recruitment of macrophages towards sites of particle deposition and inflammation.

## Results

### Lactate dehydrogenase (LDH) release from L-2 cells following treatment with fine & nanoparticle carbon black and titanium dioxide

**Figure 1:**
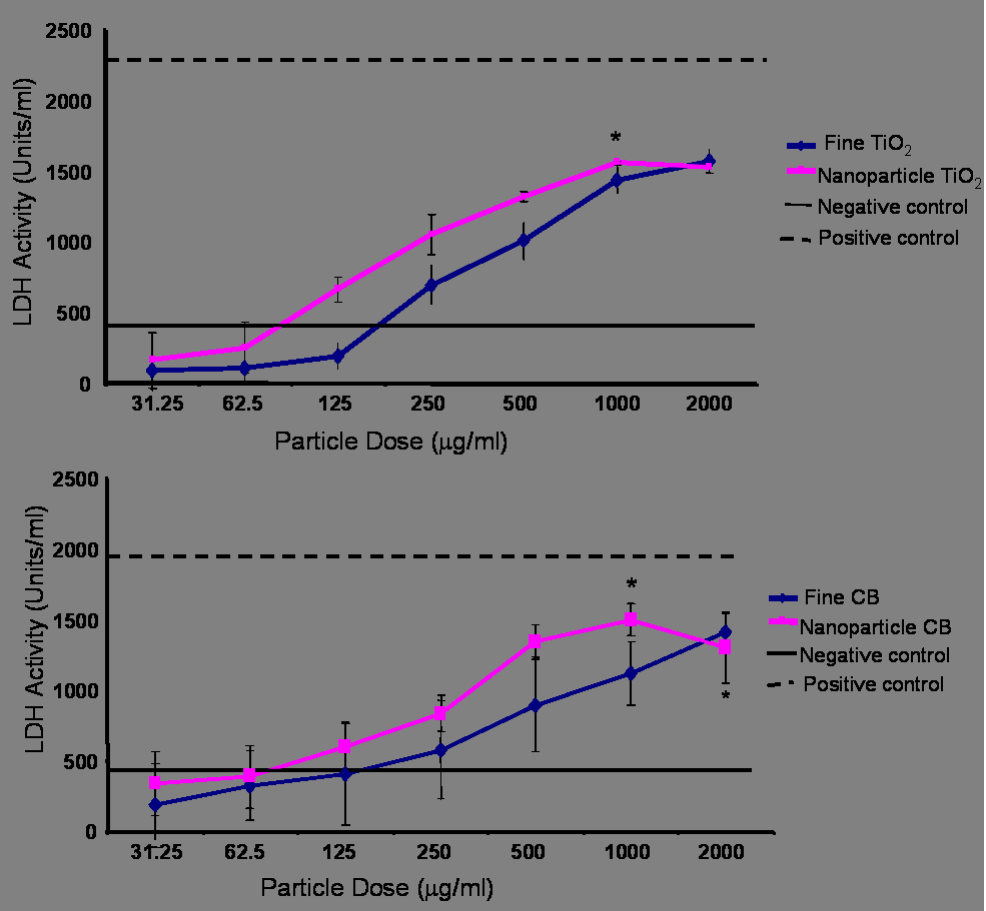
LDH release from L-2 cells following exposure to varying doses of fine and nanoparticle TiO2 (a) and carbon black (b) for 24 hours. The positive control (cells lysed with Triton X-100) and the negative control (untreated cells) are also shown. All values are the mean of three experiments conducted in triplicate ± SEM. The asterisk denotes a significant difference of the effect of both types of nanoparticles from the negative control (* p < 0.05).

Figures 1(a) and 1(b) show that LDH release from the L-2 cells increased in a dose dependant manner upon treatment with all particle types until the highest release induced by nanoparticle TiO_2 _and carbon black was reached at a particle concentration of 1 mg/ml. Maximal release of LDH following treatment with fine TiO_2 _and carbon black occurred at a particle concentration of 2 mg/ml. The 1 mg/ml treatments of nanoparticle carbon black and TiO_2 _induced a significant increase in LDH release from L-2 cells compared to the negative control (p < 0.05). Nanoparticle carbon black also resulted in a significant increase in LDH release (p < 0.05) at a particle concentration of 2 mg/ml. High doses of both types of fine particles were also observed to induce similar increases in LDH release but this was not statistically significant. However, maximal LDH release induced by particle treatments was noted to be lower than that observed following cell lysis with Triton X-100 (positive control) possibly due to high particle concentrations interfering with the assay. The negative control in these experiments (untreated cells) showed minimal LDH release. Low doses of particulates (31–500 μg) did not appear to show any significant size, composition or surface area-related differences in response.

### Concentration and time course studies with zymosan activated serum (ZAS) and J774.2 macrophages

**Figure 2:**
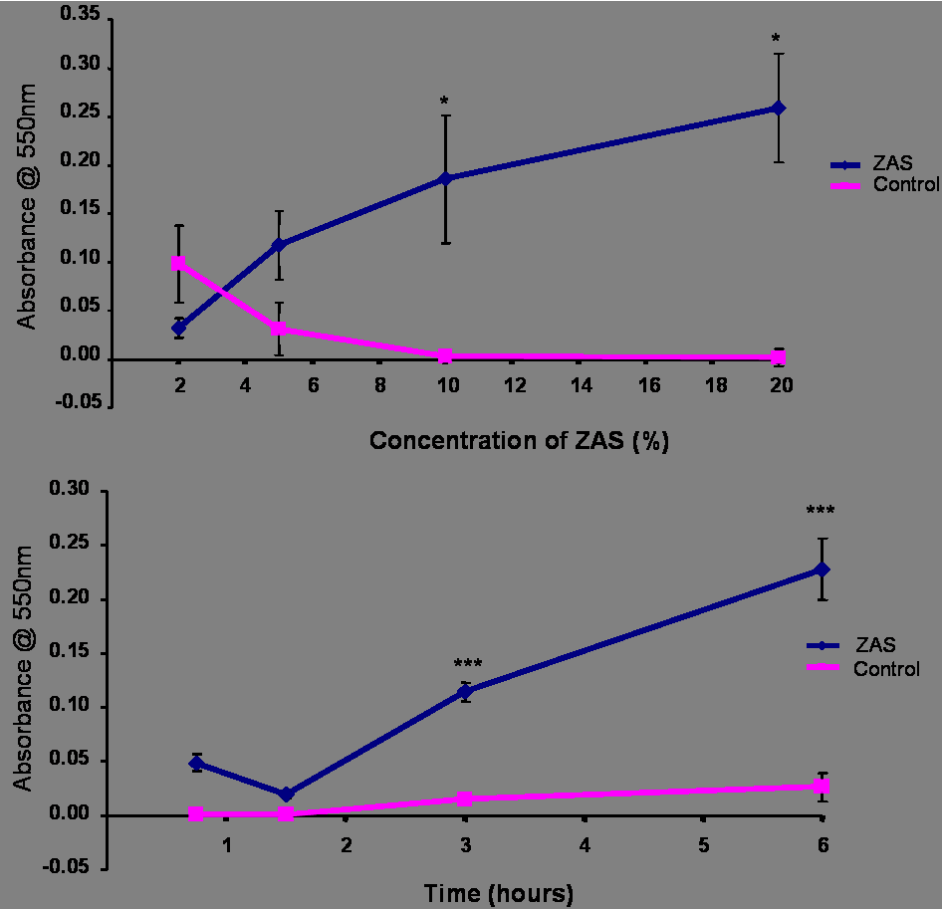
(a) The effect of varying concentrations of ZAS on macrophage migration compared to a negative control of non-activated serum. (6 hour incubation time). Asterisks denote a significant difference from the control (* p < 0.05). (b) A time course examination of macrophage migration towards 10% ZAS compared to a negative control of non-activated serum. Asterisks denote a significant difference from the control (*** p < 0.001).

Figure 2(a) shows the effects of varying concentrations of ZAS on macrophage migration following a 6 hour incubation period. The graph shows that the absorbance increases as the concentration of ZAS increases from 2 to 20% indicating increased numbers of macrophages migrating into or through the polycarbonate filter. Significant increases in macrophage migration were observed at both the 10% and 20% dilutions of ZAS. This was compared to the negative control of foetal bovine serum (FBS) incubated with sterile saline, which showed no significant increases in macrophage migration.

Figure 2(b) shows a time course study of macrophage migration towards 10% ZAS. The results indicate that the ZAS induced significant increases (p < 0.001) in macrophage migration at both the 3 hour and 6 hour time points. The highest level of macrophage migration was found at the 6 hour time point where migration was found to be 8-fold greater than that of the negative control. The negative control (FBS incubated with sterile saline) did not display any potential for macrophage migration and this may serve as an indicator that random macrophage migration, chemokinesis, was minimal.

### Macrophage migration stimulated by conditioned medium from L-2 Cells exposed to TNFα

**Figure 3:**
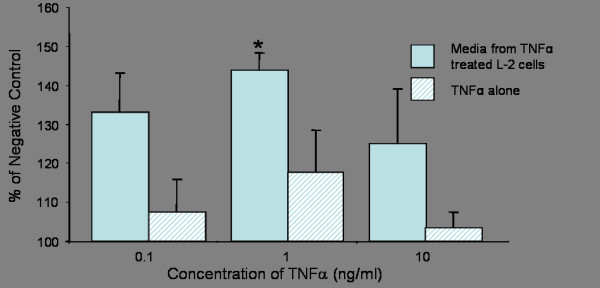
The chemotactic effect of conditioned medium from L-2 cells exposed to varying doses of TNFα compared with TNFα alone. Asterisks denote a significant change from the cell free TNFα treatment (* p < 0.05). Graph shows the result of one experiment with three replicates.

L-2 cells were exposed to TNFα in order to induce the secretion of chemotactic molecules (Figure 3). Supernatants from cells treated with TNFα at a concentration of 1 ng/ml, induced significant macrophage migration (p < 0.05) compared to the negative control (conditioned medium obtained from untreated cells). Macrophage migration was also noted at both the lower and higher TNFα concentrations although this was not significant. The other negative control, TNFα alone, at comparable concentrations to those used for cell treatments, induced modest macrophage migration.

### Macrophage migration stimulated by conditioned medium from L-2 Cells exposed to fine and nanoparticle carbon black and TiO_2_

**Figure 4:**
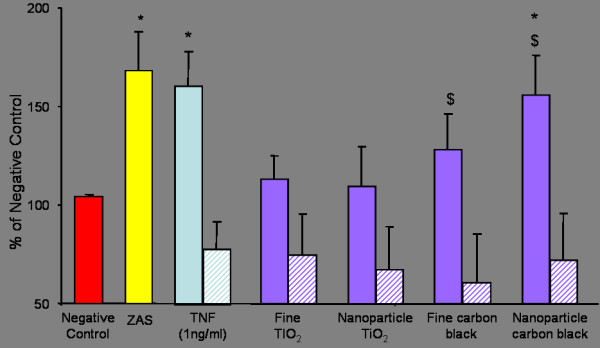
Macrophage migration induced by L-2 cell supernatant following treatment with 125 μg/ml fine and nanoparticle TiO2 and carbon black (striped bars indicate cell free treatments). ZAS and conditioned media from L-2 cells treated with TNFα (1 ng/ml) represent positive controls. Asterisks denote a significant increase compared to the negative control (* p < 0.05). Significant increases from the appropriate cell-free treatments are denoted by a dollar ($ p < 0.01).

When compared to the negative control (conditioned medium obtained from untreated cells), nanoparticle carbon black treatment of L-2 cells generated a conditioned medium that induced a significant increase (p < 0.01) in macrophage migration (Figure 4). In comparison, conditioned medium from L-2 cells treated with fine TiO_2_, CB and nanoparticle TiO_2 _did not induce any significant increases in macrophage migration. It should also be noted that both fine and nanoparticle carbon black treatment of L-2 cells did induce significant increases in macrophage migration when compared to another negative control; medium incubated with the particles alone (p < 0.01 and p < 0.001 for fine and nanoparticle carbon black respectively).

### SDS-PAGE electrophoresis of conditioned medium from L-2 cells treated with a sub-toxic dose of nanoparticle carbon black

**Figure 5:**
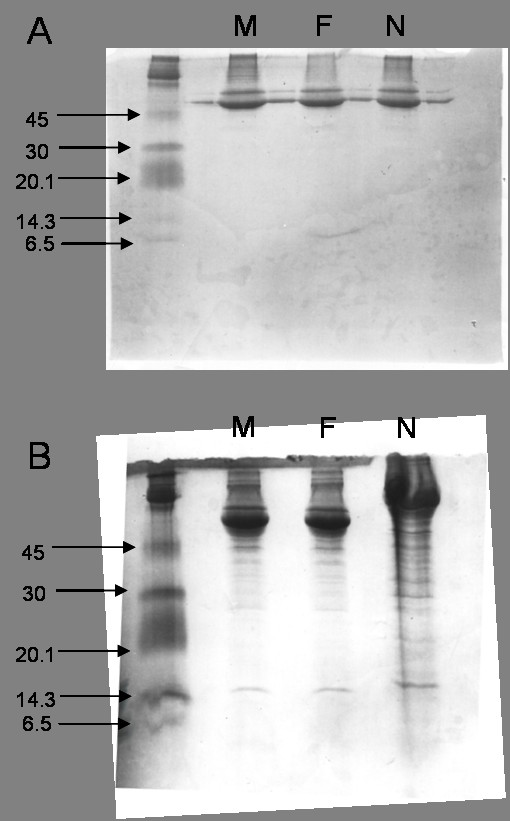
SDS-PAGE gels of conditioned medium from L-2 cells treated with 125 μg fine or nanoparticle carbon black for 24 hours. The negative control is represented by cells treated with medium only (M). Gel A was run under non-reducing conditions and Gel B was run under reducing conditions via the addition of DTT. Marker bands and corresponding molecular weights (kDa) are indicated at the left hand side of the gels. M = Medium Only, F = Fine carbon black treatment, N = Nanoparticle carbon black treatment

A non-reducing SDS-PAGE gel was used to separate the protein components of the conditioned medium obtained from L-2 cells treated for 24 hours with either fine or nanoparticle carbon black (125 μg/ml). Cells were also incubated with serum-free medium for 24 hours and this conditioned medium was run together with the two other treatments as a negative control. As can be seen from the gel (Figure 5a), only protein bands corresponding to very high molecular weights were identifiable in all treatments used.

Figures 5b shows conditioned medium obtained from similar particle treatments run under reducing conditions. As can be seen from the gel, an increased number and density of protein bands are observed in the lane containing the conditioned medium obtained from cells that have been treated with nanoparticle carbon black. Bands are clearly noted around the 14 kDa weight and the density of this band is increased in the lane containing the conditioned medium from the nanoparticle treated cells. The negative control in both of these gels, conditioned medium obtained from untreated cells, showed minimal protein banding only under reducing conditions.

### Chemotactic activity of size fractions of conditioned medium obtained from L-2 cells treated with a sub-toxic dose of nanoparticle carbon black

**Figure 6:**
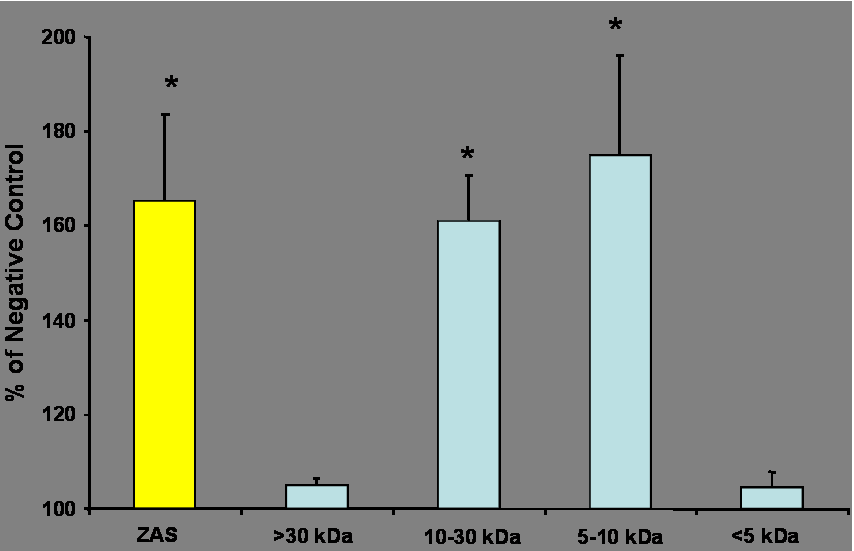
Macrophage migration induced by centrifuge fractions of conditioned medium from L-2 cells treated with nanoparticle carbon black (125 μg/ml). The positive control is indicated by the yellow bar representing migration towards ZAS. The negative control was conditioned medium obtained from untreated cells. Asterisks denote a significant increase compared to the negative control (* p < 0.05).

The conditioned medium generated by nanoparticle carbon black treatment (125 μg/ml) of L-2 cells was size-fractionated by centrifugation through size selective filters. It was found that the fraction sizes of 5–10 kDa and 10–30 kDa both induced a significant increase in macrophage migration (p < 0.01) when compared to the negative control (conditioned medium obtained from untreated cells). The other two fractions of 0–5 and >30 kDa did not induce a significant increase in macrophage migration (figure 6).

## Discussion

Combustion-derived nanoparticles have been implicated in the adverse effects of ambient particulate air pollution (reviewed in [1]) In addition, the toxicological impact of nanoparticles has now come under increased scrutiny due to the emergence of a relatively new technological discipline; nanotoxicology. Studies have shown that nanoparticles can be more toxic than an equivalent mass dose of fine particles comprised of the same material [10,11,25,26] The increased toxicity of nanoparticles can be attributed to several factors that depend on the nanoparticle under consideration, but for low toxicity, low solubility particles such as those used in the present study. the larger surface area of the particles and resulting oxidative stress appears to be the dominant property [1,27]. In this study, the effects of particle treatments on type II cells and their potential to induce macrophage migration is investigated.

When treating L-2 cells with the particles used in this study, the concentration of LDH detected in the conditioned medium increased in a dose dependant manner. Maximal LDH release was detected at doses of 1–2 mg/ml of all particles tested. These high doses would never be attained in the lungs under plausible exposure conditions but were used in the study for completeness. The nanoparticles demonstrated a non-significant trend of greater toxicity towards the cells compared to the fine particles. This was indicated by significant increases in LDH concentrations following treatment with both types of nanoparticles. While these results may provide an accurate representation of particle cytotoxicity by measuring LDH concentrations, it is possible that LDH may have been adsorbed on to the surface of the particles following release from the cells. Desai and Richards [28] and Jones *et al*., [29] both noted how different types of biological molecules could be adsorbed on to the surface of inorganic and mineral dusts. Brown *et al*., [30] also showed extensive binding of Bovine Serum Albumin (BSA) to the surface of nanoparticle carbon black. This may occur when measuring LDH concentrations, especially when taking into account the large surface area of the nanoparticles and the potential for protein adsorption.

Zymosan activated serum has been used as a positive stimulatory agent to induce macrophage chemotaxis in numerous studies with particles [31-33]. Zymosan A is a polysaccharide extracted from yeast cell walls when added to serum, activates the complement system generating large quantities of the macrophage chemoattractant C5a In this study, the J774 macrophages migrated towards ZAS in a dose-dependant manner with significant increases in macrophage migration being observed at ZAS concentrations of 10% and 20% (p < 0.001). Therefore, it was decided, based upon a review of the relevant literature and these results, that 10% ZAS would be an adequate concentration to use as a positive control in future studies using the chemotaxis chamber.

Other macrophage chemotaxis studies have routinely allowed at least 3 hours incubation time to allow macrophages to complete shape change and migration [31-34]. While 3 hours incubation appeared to allow for a significant level of macrophage migration to take place, our results indicated that 6 hours would provide more opportunity for the macrophages to migrate through the filter. This decision took into consideration the fact that future migration studies would be testing a very dilute conditioned medium and as such, macrophages responses could possibly be slower than those observed with Zymosan activated serum.

Prior to conducting particle treatments on L-2 cells, a preliminary experiment was conducted to determine if stimulation by a pro-inflammatory cytokine such as TNFα could induce the type II cells to release macrophage chemoattractants. A significant increase in macrophage migration was observed when the L-2 cells were treated with TNFα at a concentration of 1 ng/ml for 6 hours. A similar macrophage response was not observed in the cell-free supernatants i.e. TNFα at equivalent concentrations that had been incubated for 6 hours in cell-free wells. Although increased migration was observed at TNFα concentrations of 0.1 and 10 ng/ml this was not statistically significant. The 10 ng/ml TNFα concentration may be expected to induce a dose-dependant increase in migration but it is possible that the TNFα concentration could be approaching toxic levels and that this may have actually inhibited migration. The results from this experiment indicate that the L-2 cells were capable of releasing macrophage chemoattractants following pro-inflammatory stimulation. The fact that similar increases in macrophage migration were not observed with the TNFα alone indicated that residual TNFα itself in the medium could not explain the chemotactic response and that chemoattractants released by the L-2 cells into the conditioned medium were responsible. These results correlate well with those published by Standiford *et al*., [35], Paine III *et al*., [23] and O'Brien *et al*., [14] who reported that TNFα is able to induce the secretion of chemoattractants from type II epithelial cells. Studies have also shown that IL-9 also induces the release of chemoattractants from bronchial epithelial cells [36]. However, we chose TNFα as the stimulant since it has been shown to be present in the BAL fluid of particle treated animals in many *in vivo *studies [21,37,38] as well as being released by cells *in vitro *after particle treatment [19,39].

The results obtained from the LDH experiments indicated that 125 ug/ml of particles would act as a suitable sub-toxic dose for further investigation. L-2 cells were treated at that dose with four different types of particles for 24 hours and the conditioned medium that was generated was tested for potential to induce macrophage migration. The nanoparticle carbon black treatment of the L-2 cells generated a conditioned medium that induced a significant increase in macrophage migration compared to the negative control, whereas none of the other particle treatments had a significant effect on macrophage migration. This would indicate that the carbon black nanoparticles, as well as causing increased cytotoxicity at high dose, are able to stimulate the release of macrophage chemoattractants when exposed to L-2 cells at sub-toxic doses. Cell-free treatments were also conducted to determine whether any soluble substances present on the surface of the particles had the potential to directly induce macrophage migration but no such migration was stimulated. This took into consideration studies by Milanowski *et al*., [34] which showed macrophage chemotaxis towards bacterial and fungal products extracted from organic dust samples. In fact, the migration towards media treated with particles was observed to be slightly lower (although not significantly lower) than the control. This may suggest that the particles may bind or inactivate factors in the media that stimulate cell migration or that they release factors that inhibit migration.

Two nanoparticles were used in these studies, TiO_2 _and carbon black, but only the carbon black showed any effect in stimulating the release of chemotaxins. This is in accord with previous findings that fine TiO_2 _was least inflammogenic of a panel of four nanoparticle types, including nanoparticle carbon black, and had least surface free radical activity as measured in a plasmid DNA scission assay [40]. Although both are classified as low toxicity low solubility particles, nanoparticle carbon black has a much greater surface area than nanoparticle TiO_2 _and this may be a major factor in generation of free radicals *in vivo *[41].

Following the experiments using four different particle types, it was decided that further experiments should focus upon the effects of the nanoparticle carbon black on the L-2 cells and the elucidation of the products that were released from the type II cells as a result of particle exposure. SDS-PAGE gels of conditioned medium obtained from L-2 cells treated with 125 μg of either fine or nanoparticle carbon black were run in non-reducing and reducing conditions. In non-reducing conditions, no bands were visible below the 45 kDa molecular weight. However, with the addition of the reducing agent DTT, a higher number of protein bands were noted in the nanoparticle carbon black conditioned medium compared to the medium obtained from the fine carbon black and untreated cells. This indicates that an increased number and concentration of proteins were released from the type II cells into the conditioned medium as a result of the nanoparticle carbon black exposure. One or more of these proteins may account for the increased macrophage chemotactic activity in conditioned medium obtained from such cells. The increased bands were present in the molecular weight range of approximately 14 – 35 kDa. A subsequent experiment where the conditioned medium was centrifuged through molecular weight filters and then tested for chemotactic activity indicated that the substance(s) that induced macrophage migration was located in the 5 – 30 kDa weight range. This is based on the observation that the 5–10 kDa and 10–30 kDa fractions of the conditioned medium induced a significant increase in macrophage migration compared to the negative control. A number of chemotactic proteins such as MCP-1, MIP-1, MIP-2 and rantes are known to be located within these size ranges [42].

There are important points to take note of when drawing comparisons between *in vitro *studies and the dosimetry of environmental particle exposure in individuals. Obviously, exposure to a given particulate can vary depending upon a person's occupation, location, method of travel, behaviour etc. Using a miners group as a model, however, Kuempel *et al*., [43] estimated that a coal miner will inhale >350 g of coal dust over a lifetime, and that around 40 g of this will be deposited in the alveolar region of the lung. This figure is not applicable to most people, but draws attention to the potential particle exposure which can occur in a given occupation or workplace. This is a chronic exposure versus the acute exposure used in our study but is important nonetheless. Impaired clearance and protracted exposure of epithelial cells to particles are key factors when attempting to identify the reasons behind the increased toxicity induced by nanoparticles. Although some of the doses used in this study may not be physiologically relevant, they can provide some idea of how type II cells may react in response to acute and, depending on clearance efficacy, sub-chronic exposures to particles.

The phenomenon of rat lung overload in response to high exposures to low toxicity low solubility particles includes, amongst other important pathological sequelae, failure of clearance with subsequent retention of particles [44]. Under conditions of overload there is greatly enhanced contact between particles and epithelium, due to the high rate of particle deposition, exceeding the macrophage's capacity to phagocytose them [45]. Overload is driven by surface area [46] and so the findings described here provide a mechanism that may help to explain the retention of particle-loaded macrophages in the lung periphery during overload. By this mechanism, the extensive interaction between particle surfaces and epithelial cells characteristic of overload would cause release of chemotaxins that would 'attract' the particle-loaded macrophages to the alveolar region. This gradient would directly compete with the normal chemotactic gradient that would draw such macrophages up the respiratory tract for muco-ciliary clearance. Renwick *et al*., [26] demonstrated that the same types of nanoparticles used in this study, induced an increase in the potential for macrophages to migrate towards a positive chemoattractant, ZAS. In this manscript we have shown that these particles also possess the ability to induce type II epithelial cells to release elevated levels of macrophage chemoattractants. The chemoattractants can stimulate two events, the first being the migration of macrophages towards epithelial cells exposed to particles, as in the case of epithelial cell exposure to nanoparticle carbon black. This event will recruit cells and promote inflammation, but may potentially decrease subsequent clearance if the signal does not subside. In the second instance, a chemotactic gradient may stimulate removal of phagocytic cells out of the lung via the mucociliary escalator, promoting particle clearance. The data shown here demonstrates that nanoparticle carbon black was the most potent tested at eliciting chemotaxin release. This may suggest that such nanoparticles could reverse the normal chemotactic gradient and prevent clearance leading to overload at a lower mass burden than larger particles and this is in fact, amply supported by data (reviewed in [47]). These are conflicting interpretations of particle effects and require further work to determine the exact pathways governing particle clearance.

## Conclusion

The regulation and stimulation of leukocyte recruitment in the lung is an important factor in the regulation of inflammation and several regulatory mechanisms exist. The response of macrophages to paracrine signals appears to be an important driver for inflammation and macrophages respond to chemotactic signals from neutrophils [31], type II cells [14] and from other macrophages [48]. Other studies have focussed on the inhibition of macrophage chemotaxis induced by molecules such as the macrophage migration inhibitory factor (MIF), described by Hermanowski-Vosatka *et al*., [49] which inhibits macrophage movement. However, as mentioned previously, several studies have demonstrated that type II cells can secrete a wide range of pro-inflammatory mediators capable of inducing macrophage migration to sites of inflammation. It is likely that, due to the large surface area of carbon black nanoparticles and their subsequent ability to induce oxidative stress [11], there is signalling for expression of genes for chemotaxins by the type II cells. This may indicate an adaptive response of lung epithelial cells in contact with deposited particles which would aid in the rapid recruitment of inflammatory cells to the sites of particle deposition, and the subsequent removal of the particles by phagocytic cells such as macrophages and neutrophils.

Obvious future studies in this area could pursue the identity of the substance(s) released by the type II cells in response to particle exposure. This study indicates that the factors inducing macrophage migration are in the molecular weight range between 5 and 30 kDa. However, due to the wide range of substances released by the type II cells, it is likely that no one single substance is responsible for inducing macrophage recruitment and, as such, further experiments in this area may involve deciphering the complex mixtures of chemotactic molecules released by the type II cells to ascertain their specific cellular targets. Synergistic or potentiative interactions between the chemotactic molecules should also be taken into consideration. Other investigations may attempt to examine the effects of other forms of particles as well as various components of particulate air pollution.

## Materials and methods

### Chemicals and reagents

L-Glutamine (200 mM); penicillin-streptomycin (1000 μg/ml) and foetal bovine serum (FBS) were obtained from Invitrogen, UK. RPMI 1640 (without L-glutamine), Zymosan A and Bovine Serum Albumin (BSA) were obtained from Sigma Chemicals Company, Dorset, UK. The Rapi Diff 2 (Romanowsky) stain set was obtained from Raymond A Lamb, London. All other chemicals and reagents were purchased from Sigma Chemicals Company, Dorset, UK unless otherwise stated.

### Culture of L-2 type II epithelial cell line

The rat alveolar type II cell line L-2 was obtained from the European Collection of Animal Cell Cultures (Salisbury, England). The cells were grown in 25 cm^2 ^tissue culture flasks in RPMI 1640 medium supplemented with 1% L-glutamine, 1% penicillin/streptomycin and 10% heat inactivated FBS. Cultures were incubated in a humidified incubator at 37°C/5% CO_2_. Cell counts were performed using 0.3% trypan blue exclusion (1:1 dilution with cells) and an improved Neubauer haemocytometer. Cells were removed from the flask by adding 1 × trypsin/EDTA in Hanks balanced salt solution (HBSS) and incubating for 5 minutes, then centrifuged at 900 g for 2 minutes and resuspended in RPMI 1640 supplemented with 10% FBS.

### Culture of J774.2 macrophage cell line

The adherent murine monocytic-macrophage cell line J774.2 was obtained from the European Collection of Animal Cell Cultures (Salisbury, England). The cells were grown in 25 cm^2 ^tissue culture flasks in RPMI 1640 medium supplemented with 1% L-glutamine, 1% penicillin/streptomycin and 10% heat-inactivated FBS. Culture flasks were stored in a humidified incubator at 37°C and 5% CO_2_. Cell counts and viability were assessed using an improved Neubauer haemocytometer and trypan blue exclusion. All cells that were found to be non-adherent in the culture flasks were discarded by washing prior to use.

### Particles

The particles used in these experiments were fine carbon black (H. Haeffner & Co Ltd., Chepstow, UK), fine titanium dioxide (Tioxide Ltd.) and nanoparticle carbon black and titanium dioxide (Degussa, UK). The particle composition is identical for both types of carbon black and both types of TiO_2 _respectively. However, the varying diameter and surface area of the particles are described in Table 1.

**Table 1:** Mean diameter and surface area of particles used

**Particle Type**	**Mean Diameter (nm)**	**Surface Area (m^2^/g)**
Fine TiO_2_	250.0	6.6
Nanoparticle TiO_2_	29.0	49.78
Fine Carbon Black	260.2	7.9
Nanoparticle Carbon Black	14.3	253.9

Particle data taken from Renwick *et al*., [26]

### Cell treatments

Cells were seeded at 40,000 cells per well in a 96 well plate and incubated for 24 hours in RPMI 1640 supplemented with 10% FBS. Serial dilutions (62.5 – 2000 μg/ml by mass dose) of each type of particle were prepared in serum-free RPMI and sonicated in a water bath sonicator for 5 minutes before use. After 24 hours, the medium was removed from the cells and replaced with appropriate concentrations of particle before incubation for a further 24 hours. Following particle treatments, the medium was removed from the cells and centrifuged for 30 minutes at 15000 g to remove the particles. The supernatants were removed and stored in separate tubes at -80°C for use in the LDH assay.

### LDH assay

Positive control samples for the LDH assay were prepared by lysing cells with 50 μl of 0.1% Triton X-100 dissolved in phosphate buffered saline (PBS). The solutions were then centrifuged at 11,000 g for three minutes and 10 μl of the supernatant was used as a positive control to indicate total releasable LDH. A negative control was obtained by incubating the cells with serum-free medium. To create a standard curve, pyruvate standards were prepared (using distilled H_2_O and 1 mg/ml NADH dissolved in 0.75 mM sodium pyruvate), ranging in concentrations equivalent to 0 – 2000 Units/ml of LDH. The standards were transferred (60 μl) in triplicate, to a 96-well plate. Into all of the remaining wells of the plate, 50 μl of NADH was added. The samples were incubated at 37°C/5% CO_2 _for 5 minutes.

The test and control samples (10 μl) were added to the relevant wells on the plate containing the NADH solution. The plate was incubated at 37°C, 5% CO_2 _for 30 minutes. Following incubation, 50 μl of 2, 4-dinitrophenylhydrazine (0.2 mg/ml dissolved in 1 M HCl) was added to all of the wells and the plate was left for 20 minutes at room temperature. Sodium hydroxide (50 μl of 4 M concentration) was then added to each well and the plate was left at room temperature for 5 minutes. The plate was shaken for 5 seconds and the absorbance of all the wells was measured at 540 nm with a Dynex MRX microplate reader.

### Macrophage chemotaxis assay

The chemotaxis apparatus utilised in these studies was a re-usable 96-well Neuroprobe chemotaxis chamber (Receptor Technologies, UK). Each sample (30 μl) was loaded, in triplicate, into the bottom wells of the chamber. A Neuroprobe polycarbonate filter (pore size 5 μM) was inserted between the layers. J774.2 macrophages (2 × 10^5^) in 200 μl of serum-free RPMI 1640 were added to the top of each well. The chamber was incubated at 37°C in 5% CO_2 _for 6 hours and the filter was removed and washed three times with PBS on the upper side to remove non-migrated macrophages. The filter was stained with a Romanowsky (Diff-Quick) stain. The optical density of each well on the filter was read at 540 nm in a Dynex multiwell plate reader. Increasing absorbance correlates with the increasing number of macrophages moving through the filter.

### Zymosan activated serum (ZAS)

ZAS is a known activator of C5a in blood serum and, as such, has been utilised in numerous studies as a positive control in chemotaxis assays. A stock solution of Zymosan A in saline (10 mg/ml) was prepared and sonicated for 5 minutes. The stock Zymosan solution (100 μl) was added to 900 μl of FBS. To prepare a control solution, 100 μl of sterile saline was added to 900 μl serum. Both solutions were incubated in a shaking water bath at 37°C for 2 hours and then at 56°C for 30 minutes. The solutions were centrifuged for 5 minutes at 2000 g then aliquoted and stored at -80°C until use. For use in the chemotaxis assay, both solutions were diluted to the appropriate concentration using serum-free RPMI 1640.

To determine the optimum incubation times and ZAS concentrations for use in the chemotaxis assay, a solution of ZAS (1 mg/ml) and a negative control were prepared as detailed above. For the concentration response experiments, both solutions were diluted to 2%, 5%, 10% and 20% with RPMI 1640 medium. Solutions were then tested using the chemotaxis protocol as described above. For the time course experiments, 10% ZAS solution was prepared and tested in the chemotaxis chamber as detailed above. The cells were incubated in the chamber for 45 minutes, 90 minutes, 3 hours or 6 hours before the filter was removed and stained.

### Exposure of L-2 cells to TNFα

L-2 cells were seeded in a 96-well plate at a cell count of 40,000 cells per well and incubated overnight. Rat TNFα (Biosource, UK) was prepared at three different concentrations; 0.1, 1 (positive control) and 10 ng/ml using sterile PBS containing 0.1% bovine serum albumin. The medium from the L-2 cells was removed and replaced with 200 μl of TNFα at the required concentration. TNFα was also added at an equivalent concentration to wells that did not contain any L-2 cells in order to provide a cell-free comparison. Negative controls were prepared by adding serum-free RPMI to both the L-2 cells and the cell-free wells. The plate was incubated in a humidified incubator at 37°C and 5% CO_2 _for 6 hours. Following incubation, the supernatants were removed and tested for potential to induce macrophage migration using the chemotaxis assay protocol as described previously.

### Exposure of L-2 cells to particles

A 96-well plate was seeded with L-2 cells (40,000 cells per well) and incubated overnight in a humidified incubator at 37°C and 5% CO_2_. Suspensions of fine and nanoparticle carbon black and titanium dioxide were prepared in serum-free RPMI at a concentration of 125 μg/ml. The suspensions were sonicated for 10 minutes prior to use. A 1 ng/ml TNFα solution was also prepared in 0.1% BSA in PBS to be used as a positive control. The serum-free particle suspensions and TNFα were added to the relevant wells in the 96-well plate with L-2 cells. A duplicate cell-free 96-well plate was also prepared using identical solutions of TNFα and serum-free particle suspensions. Both 96-well plates were subsequently incubated in a humidified incubator at 37°C and 5% CO_2_. After 24 hours incubation, the two plates were removed and the supernatants were extracted and stored in eppendorf tubes. The eppendorf tubes were centrifuged at 15,000 g for 15 minutes and the supernatants transferred to fresh tubes. The samples were stored at -80°C prior to analysis.

### Preparation of L-2 conditioned medium

Trypsin/EDTA in HBSS (1×) was added to a flask of L-2 cells at approximately 80% confluence and incubated for 5 minutes at 37°C/5% CO_2_. Serum-free RPMI culture medium (10 mls) was added to the cells and the cells were centrifuged at 900 g for 2 minutes. The supernatant was decanted and the cells were resuspended in normal culture medium. Cell counts were assessed and adjusted to 1 million cells per ml. The cells were seeded in a 6 well plate at 1 million cells per well and incubated overnight.

To prepare particle suspension, fine and nanoparticle carbon black was suspended in serum-free RPMI at a concentration of 1 mg/ml and sonicated for 10 minutes before diluting to 125 μg/ml. The serum-free particle suspensions (200 μl) were added to the 6-well plate and incubated for 24 hours. Cells were also incubated with serum-free RPMI for 24 hours. Following incubation, the conditioned medium was removed and centrifuged at 15,000 g for 15 minutes and the supernatants were pooled and stored at -80°C until use.

### SDS-PAGE gel electrophoresis of L-2 conditioned medium

A 15% separating gel was prepared by mixing 23% distilled H_2_O, 50% acrylamide mix, 25% Tris HCl (1.5 M, pH 8.8), 1% sodium dodecyl sulphate (SDS, 10%) and 1% ammonium persulphate (APS, 10%). TEMED was added at a concentration of 0.04% immediately prior to pipetting the gel into the casting apparatus. A stacking gel was prepared by mixing 68% H_2_O, 17% acrylamide mix, 12.5% Tris HCl (0.5 M, pH 6.8), 1% SDS (10%) and 1% APS (10%). TEMED was added at a concentration of 0.1% immediately prior to adding the gel to the separating gel in the casting apparatus. Immediately following addition of the stacking gel to the casting apparatus, 1 μl of bromophenol blue was added to the stacking gel.

To prepare the samples, 20 μl of L-2 conditioned medium was pipetted into an eppendorf. For the non-reducing gel (Figure 5a), 30 μl of sample buffer was added to the conditioned medium. For the reducing gel (Figure 5b), 27 μl of sample buffer and 3 μl of dithiothreitol (DTT) was added to each of the samples. Both sets of samples were then incubated on a heating block; 1 minute incubation for non-reducing samples and 2 minutes for reducing samples. After heating, the samples were immediately stored on ice.

Once cast, the gels were transferred to an electrophoresis tank and immersed in 1 × buffer. Samples (20 μl) as well as coloured molecular weight markers were added to the gels and the electrophoresis was run at 40 mA for 45 minutes. Both gels were stained with the Coomassie blue stain overnight and destained by microwaving (3 × 1 minute) in fresh distilled water.

### Differential centrifugation of L-2 conditioned medium

Conditioned medium, obtained from L-2 cells treated with nanoparticle carbon black according to the protocol above, was separated according to molecular weight using Vivaspin 2 centrifuge filters operated according to manufacturers' instructions. A 30 kDa filter was used first, followed by a 10 kDa filter and finally a 5 kDa filter. Supernatants were stored at -80°C until tested for potential to induce macrophage migration using the chemotaxis protocol outlined above. The negative control in this experiment was conditioned medium obtained from cells that had not been treated with any particles

### Statistical analysis

Results are expressed as the mean ± SEM. All figures are the results of three separate experiments with three observations in each unless otherwise stated. Statistical analysis was accomplished by a one-way ANOVA with Tukey's multiple comparison.

## Competing interests

The author(s) declare that they have no competing interests.

## Authors' contributions

PB aided in the conception of the study, drafted the manuscript and performed all assays. ACB participated in the conception and design of the study. KD participated in the design of the study and scientific contribution to the manuscript. JM participated in critical assessment and scientific contribution to the manuscript. VS supervised the study and participated in the data analysis and drafting of the manuscript. All authors have read and approved the final manuscript.
